# Anti‐Thrombotic Effect of Hemp Seed Peptides in Zebrafish by Dual Regulation of vegfr1 Signaling and Caspase‐3‐Mediated Apoptosis

**DOI:** 10.1002/fsn3.70756

**Published:** 2025-08-01

**Authors:** Jie Shi, Jingyi Ge, Zhenghai Zhang, Lianhui Wei, Yan Dong, Guowei Li, Qingli Yang, Jing Pan, Yanru Ji

**Affiliations:** ^1^ Daqing Branch of Heilongjiang Academy of Sciences Daqing Heilongjiang China

**Keywords:** antithrombotic activity, caspase‐3 apoptosis, hemp seed peptides, vegfr1 signaling, zebrafish model

## Abstract

This study aimed to investigate the antithrombotic activity of hemp seed peptides derived from trypsin hydrolysates and elucidate their molecular mechanisms using a ponatinib‐induced zebrafish ( *Danio rerio* ) thrombosis model. Through ultrafiltration and LC–MS/MS analysis, we identified 1741 peptide sequences (< 3 kDa), with 67% distributed in the 0.5–1.5 kDa range. In vivo experiments demonstrated that 125 μg/mL hemp seed peptides significantly ameliorated ponatinib‐induced thrombosis by increasing 16.55% cardiac erythrocyte staining intensity, enhancing 54.8% blood flow velocity, and restoring 11.2% intersegmental blood vessel diameters. In addition, the observed anticoagulant effect may be partially attributed to upregulated expression levels of vascular endothelial growth factor receptor 1 (*vegfr1*) and down‐regulated expression levels of cysteinyl aspartate‐specific proteinase‐3 (*caspase‐3*) at the mRNA level. These findings propose hemp seed peptides as novel dietary antithrombotic agents with potential applications in functional food development.

## Graphical abstract

Characterize the thrombin‐inhibitory capacity of hemp seed peptides. Evaluate antithrombotic efficacy through hemodynamic and vascular parameters. Elucidate mechanisms via *vegfr1* and *caspase‐3* gene expression analysis.

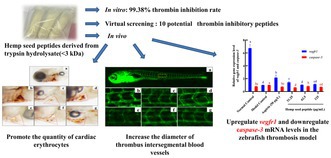

## 1 Introduction

Thromboembolism and its complications exhibit incredibly high morbidity and mortality worldwide, constituting a significant threat to human health (Klemen et al. 2020). The critical strategy of clinical prevention and treatment of thrombosis involves inhibiting coagulation functions to delay thrombus formation and extension (Hozayen et al. 2021). Heparin is a widely used anticoagulant known for its therapeutic actions, but it is always accompanied by severe side effects such as bleeding and thrombocytopenia (Li et al. 2020). Consequently, the discovery of safer anticoagulant agents as alternatives to heparin has attracted increasing attention.

Thrombin is a serine protease that plays a pivotal role in the coagulation cascade (Yu et al. 2019). In modern medicine, inhibition of thrombin activity serves as an effective means to prevent coagulation, making it an ideal target for anticoagulant therapy. Due to the abundance of functional food components that demonstrate anticoagulant properties, it is feasible to effectively regulate blood coagulation while minimizing the risk of adverse effects. Therefore, developing functional foods to prevent thrombotic diseases by targeting thrombin may represent a more advantageous approach (Schwienhorst 2006). Bioactive peptides derived from food, comprising 2–20 amino acids, are characterized by high safety and significant curative potential in nutrition and food science (Yang et al. 2021). In recent years, numerous studies have reported the development of antithrombotic peptides from various foods, including soy protein peptides (Xu et al. 2022), oyster protein peptides (Chen, Cheng, et al. 2019), and amaranth protein peptides (Sabbione et al. 2015). Zhang (2016) found that peanut protein hydrolysates prepared with alkaline have antithrombotic activity. According to reports, peptide NMEKGSSSVVSSRM isolated from seaweed can effectively prolong activated partial thromboplastin time and thus exert anticoagulant effects (Indumathi and Mehta 2016).

Hemp ( *Cannabis sativa* . L) is a well‐known herbaceous plant belonging to the Cannabaceae family, and its industry has been developing rapidly (Helstad et al. 2022). Hemp seed cake, a by‐product of oil extraction, is often underutilized and frequently discarded. Research indicated that hemp seed cake is rich in protein and highly nutritious (Leonard et al. 2020). Hemp seed peptides are products obtained through the hydrolysis of hemp seed cake protein using proteolytic enzymes, followed by special processing. These peptides exhibit diverse biological activities, including but not limited to the regulation of oxidative, anti‐inflammatory effects, reduction of blood glucose levels, and lipid‐lowering properties (Chen et al. 2023). Previously, our team discovered enhanced thrombin inhibition in trypsin‐hydrolyzed hemp seed protein (Shi et al. 2025). As a serine endopeptidase, trypsin selectively cleaves peptide bonds at the C‐terminus of lysine and arginine residues (Sun et al. 2023). These released cationic peptide fragments demonstrate targeted binding to anionic domains of coagulation factors and platelet membranes through electrostatic interactions, effectively disrupting the critical coagulation cascade and thereby suppressing thrombotic activity (Yang et al. 2025). Notably, trypsin‐mediated proteolysis of sustainable protein sources, including casein (Tu et al. 2017), *Tenebrio molitor* larvae (Chen, Jiang, et al. 2019), and blue mussel (Qiao et al. 2018), has yielded bioactive peptides with comparable antithrombotic effects, highlighting the potential of trypsin enzymatic proteolysis for developing antithrombotic functional food ingredients.

Nevertheless, the low absorption efficiency of peptides within the body presents a leading challenge, compounded by the potential loss of activity during the digestive process. In contrast, in vivo experiments are highly reliable and contribute to a better understanding of the occurrence and physiological mechanisms underlying thrombotic diseases. Meanwhile, there remains a lack of sufficient evidence to clarify that hemp seed peptide has antithrombotic activity in vivo and its underlying mechanism for treating thrombosis. Animal models are an indispensable tool to understand the underlying pathogenesis of thrombosis and assess the efficacy of novel antithrombotic agents (He, Du, et al. 2024). The transgenic zebrafish ( *Danio rerio* ) lines are available for visual research, particularly erythrocyte labeling. Besides, given the optical transparency that facilitates visualization of blood cells, vessels, and clots, zebrafish have emerged as a reliable model for thrombosis and have been widely employed in research (Griffin et al. 2024).

Therefore, the main objective of this study was to prepare highly active anticoagulant peptides from hemp seed cake and explore the antithrombotic effects using a zebrafish model. Initially, toxicity assessments were performed to determine zebrafish's maximum tolerated concentration (MTC) in response to hemp seed peptides. Subsequently, the antithrombotic efficacy of hemp seed peptides was evaluated by comparing parameters such as erythrocyte staining intensity in zebrafish cardiac, blood flow velocity, and intersegmental vascular diameter. This research is of significant importance for enhancing the added value of hemp processing by‐products and further developing functional foods to prevent thrombosis.

## 2 Materials and Methods

### 2.1 Materials

Methylcellulose (Aladdin, China); punaritine (MedChemExpress, USA); O‐dianisidine (Sigma, USA); aspirin (Bayer, Germany); ChamQ Universal SYBR qPCR Master Mix (Vazyme, China); FastKing cDNA First Strand Synthesis Kit without genomic DNA (Tiangen, China); pre‐packed magnetic bead‐based Universal RNA Extraction Kit C (Foshan Aowei, China).

AB wild‐type, albino melanin mutants, and transgenic fli1‐EGFP zebrafish (5 dpf, Hunter Biotec, China). The license number for the experimental zebrafish is SYXK (Zhe) 2022‐0004. All zebrafish experiment protocols complied with the standards established by international AAALAC certification (certification no.: 001458). We confirm that all zebrafish experiments were conducted in strict accordance with the guidelines set forth by the Institutional Animal Care and Use Committee (IACUC) Chairman of Hunter Biotechnology Inc., under approval number: IACUC‐2022‐5407‐01.

### 2.2 Methods

#### 2.2.1 Preparation of Hemp Seed Protein Hydrolysate

Hemp protein hydrolysate was prepared using a solid–liquid ratio of 1:25, adding trypsin at a concentration of 5.68% (Shi et al. 2025). The reaction was carried out at pH 8.0 and 40°C for 5 h. The enzyme was inactivated by heating to 90°C for 10 min. The supernatant was collected for further utilization after centrifugation at 5000 rpm for 15 min.

#### 2.2.2 Ultrafiltration Separation of Hemp Seed Protein Hydrolysate

FlowMem 0015 Ultrafiltration equipment (Xiamen Fumei Technology Co. Ltd., China) was used to separate the hydrolysate. The ultrafiltration membranes with molecular weight cutoffs of 3 and 5 kDa were employed for the ultrafiltration process according to Qi et al.'s method (Qi et al. 2022). The resulting filtrate was lyophilized and collected, subsequently prepared at concentrations of 0.005, 0.01, and 0.02 g/mL to assess thrombin inhibition rates. Furthermore, hemp seed peptide powder with a molecular weight below 3 kDa was utilized in zebrafish experiments. Molecular weight distributions were evaluated using High‐Performance Liquid Chromatography (HPLC) with an Agilent 1260 Infinity II system (USA), equipped with a TSKgel G2000 SWXL column (7.8 × 300 mm, TOSOH, Japan).

#### 2.2.3 Identification of Peptide Sequences by LC–MS/MS

Referring to the method proposed by Zhang et al. (2025), the sample underwent separation using an AcciaimPepMap RP‐C18 chromatography column coupled with an Easy‐nLC 1200 system (Thermo Scientific, USA). Subsequently, the peptides were analyzed on the Q‐Exactive HF‐X via a data‐dependent Top 20 approach and combined with the database of UniProt_Cannabis_sativa_reference_proteome_30194_20221124; MaxQuant Ver. 1.5.5.1 was employed for mass data identification.

#### 2.2.4 Virtual Screening

The physicochemical properties of candidate antithrombotic peptides were predicted using multiple databases (Table 1). Peptides identified by LC–MS/MS were screened against known antithrombotic peptides in BIOPEP‐UWM and published literature, and no identical matches were found.

**TABLE 1:** Databases used for physicochemical property prediction.

Physicochemical properties	Database	WebSite
Toxicity	ToxinPred	https://webs.iiitd.edu.in/raghava/toxinpred/
Allergenicity	AllerCatPro	https://allercatpro.bii.a‐star.edu.sg/
Isoelectric point (pI)	Expasy	https://web.expasy.org/compute_pi/
Hydrophobicity	ProtParam	https://web.expasy.org/protparam/
Solubility and net charge	Innovagen	http://www.innovagen.com/
Hemolytic potential	HemoPI	https://webs.iiitd.edu.in/raghava/hemopi/batch.php/

UCSF DOCK 6 was employed for structure‐based virtual screening (SBVS). Thrombin (PDB ID: 2BVR) served as the receptor protein, and its crystal structure was retrieved from the RCSB database (http://www.rcsb.org/). The three‐dimensional (3D) structures of the peptides were predicted using RDKit (https://rdkit.org). Lower docking scores (indicating favorable spatial complementarity) corresponded to higher binding affinity between ligands and thrombin (2BVR), suggesting stronger potential biological activity.

#### 2.2.5 Thrombin Inhibition Rate Detection Assay

The thrombin inhibition rate was assessed using a microplate reader colorimetric method, as described previously with some modifications (Huang et al. 2020). A total of 140 μL of fibrinogen solution and 40 μL of sample solution were added to the wells of a 96‐well microplate, then absorbance at 405 nm (*A*_SB_) was measured using an Epoch microplate reader (BioTek, USA). The absorbance was rerecorded after adding 20 μL thrombin solution at 37°C for 20 min (*A*_S_). For the blank control, 40 μL of 50 mM Tris–HCl buffer (pH = 7.4) was used as blank instead of the sample, while all other procedures remained consistent with those applied to the samples. The absorbance for the blank was read at 405 nm (*A*_CB_) and (*A*_C_). The thrombin inhibition rate was calculated according to the following formula:

$$\begin{array}{c} \text{Thrombin inhibition rate}\;\left(\%\right)=\left[\left(A_{C}-A_{\mathrm{CB}}\right)-\left(A_{S}-A_{\mathrm{SB}}\right)\right]/\left[\left(A_{C}-A_{\mathrm{CB}}\right)\right]\\ \;\times 100\% \end{array}$$

#### 2.2.6 Determination of Maximum Tolerated Concentration (MTC)

The Melanin allele mutant Albino zebrafish were randomly placed into a 6‐well plate, with 30 zebrafish in each well. Hemp seed peptides were dissolved in distilled water at 125, 250, 500, and 1000 μg/mL concentrations. After treatment at 28°C for 18 h, the MTC of the model zebrafish was determined based on the phenotypes and mortality.

#### 2.2.7 Establishment of Zebrafish Model and Drug Treatment

In this assay, 180 5 dpf zebrafish were divided into six groups: normal control, model control (ponatinib), positive control (ponatinib with 50.0 μg/mL aspirin), and hemp seed peptide group (ponatinib with peptide at 31.25, 62.5 and 125 μg/mL in distilled water). The model group was exposed to water‐soluble ponatinib, which was designated as a zebrafish vascular endothelial injury model. Each group of zebrafish was placed in a 6‐well plate for 18 h, and each well contained 3 mL water.

#### 2.2.8 Analysis of Staining Intensity in Cardiac Erythrocytes

For every group, 10 Melanin allele mutant Albino zebrafish were randomly selected, stained with O‐dianisidine for 30 min in the dark, and washed with DMSO as previously described (Yang et al. 2024). After fixation in 5% paraformaldehyde, zebrafish cardiac were photographed using a VertA1 CCD Camera (Shanghai Tusen Vision Technology Co. Ltd., China) under an SZX7 dissecting microscope (Olympus, Japan) (Xu et al. 2021). NIS‐Elements D 3.20 advanced image processing software was then employed to analyze the data. The staining intensity (SI) of erythrocytes was quantified to evaluate the efficacy of hemp seed peptides in treating vascular endothelial injury‐related thrombosis. The antithrombotic effect was calculated using the equation below (Zhu et al. 2016).

$$\text{Antithrombotic effect}\;\left(\%\right)=\left({\mathrm{SI}}_{\text{drug}}-{\mathrm{SI}}_{\text{model}}\right)/\left({\mathrm{SI}}_{\text{control}}-{\mathrm{SI}}_{\text{model}}\right)\times 100$$

#### 2.2.9 Investigation of Hemodynamic Improvement in Blood Flow Velocity

To investigate the dynamics of erythrocytes in AB wild‐type zebrafish, we employed the ZebraBlood 3.4 devices (ViewPoint Life Sciences, France) to capture hemal flow in the dorsal aorta (M. Sun et al. 2021). Subsequently, we analyzed the blood flow velocity of the zebrafish to evaluate the hemodynamic effects of hemp seed peptides on improving vascular endothelial injury‐related thrombosis.

#### 2.2.10 Evaluation of the Efficacy in Enhancing the Diameter of Thrombus Intersegmental Blood Vessels

Ten transgenic green fluorescent vascular zebrafish in each group were randomly selected for imaging under an AZ100 fluorescence microscope (Nikon, Japan). The data obtained were processed and analyzed by NIS‐Elements D 3.20 advanced image processing software to assess the diameter of the intersegmental blood vessels in zebrafish.

#### 2.2.11 Exploration of the Mechanisms Underlying Vascular Endothelial Injury and Improvement in Thrombosis

Real‐time quantitative reverse transcription PCR (RT‐qPCR) was performed to measure the gene expression levels of thrombosis‐related genes using the methods reported before (Zhu et al. 2020). Total RNA was extracted from AB wild‐type zebrafish using the Auto‐Pure32A automatic nucleic acid extraction instrument (Hangzhou Aosheng Instrument Co. Ltd., China). The concentration and purity of total RNA were determined through a Nanodrop2000 ultraviolet–visible spectrophotometer (Thermo, USA). Subsequently, 2.00 μg of RNA was employed to synthesize 20 μL of cDNA following the protocol provided by the cDNA first‐strand synthesis kit. The relative expression levels of the vascular endothelial growth factor receptor 1 (*vegfr1*) and cysteinyl aspartate‐specific proteinase‐3 (*caspase‐3*) genes were calculated by using the CFX Fluorescence quantitative PCR instrument (BIO‐RAD, Singapore), with *β‐actin* serving as an internal reference. The primer sequences are presented in Table 2.

**TABLE 2:** Primer sequences of *vegfr1, caspase‐3* and *β‐actin*.

Gene	Forward primer	Reverse primer
*vegfr1*	5′‐GAGCGATGCTCCCGTTATCA‐3′	5′‐CACAACTCCACTCTCCCTGG‐3′
*caspase‐3*	5′‐CCGCTGCCCATCACTA‐3′	5′‐ATCCTTTCACGACCATCT‐3′
*β‐Actin*	5′‐TCGAGCAGGAGATGGGAACC‐3′	5′‐CTCGTGGATACCGCAAGATTC‐3′

### 2.3 Statistical Analysis

Statistical analysis was performed using SPSS 26.0 software and expressed as mean ± standard error. Data were analyzed by one‐way ANOVA with Tukey's post hoc test, and *p* < 0.05 indicated statistical significance.

## 3 Results

### 3.1 Purification of Antithrombotic Peptides From Hemp Seed

**FIGURE 1:**
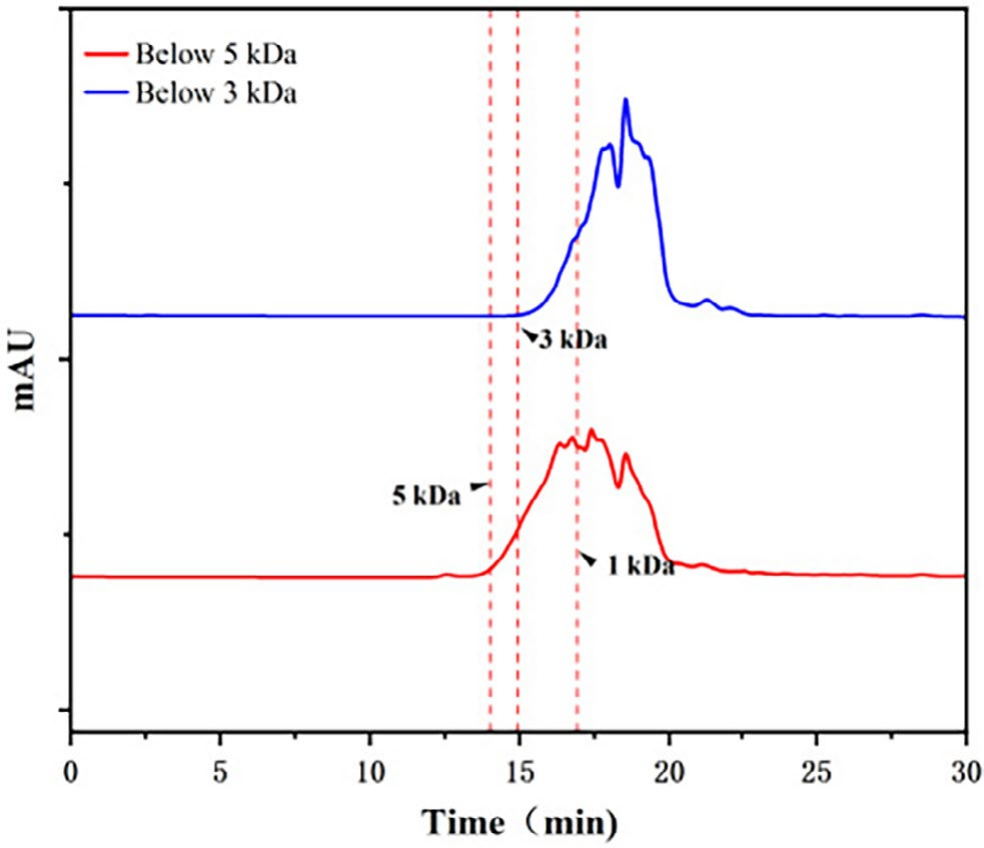
Molecular weight distribution profile of peptides.

It is widely acknowledged that the molecular weight of peptides is thought to be highly linked to their biological activity. Hemp protein hydrolysate was a mixture of peptide segments with varying molecular weights and a small amount of unhydrolyzed protein. This study used ultrafiltration membrane technology to achieve the hemp seed crude antithrombotic peptide. Molecular weight distribution profiles confirmed the effective separation of hemp seed peptides via ultrafiltration into fractions below 3 kDa and below 5 kDa (Figure 1), and the thrombin inhibition rate of these fractions was assessed. Through processes including ultrafiltration, freeze‐drying, and quantitative sampling detection, it was observed that the hemp seed peptide with a molecular weight under 3 kDa exhibited a significantly stronger thrombin inhibition rate at a concentration of 0.01 g/mL, reaching as high as 99.38%, as shown in Table 3. Furthermore, its IC_50_ value for hemp peptides was determined to be 4.55 mg/mL, lower than that of rapeseed peptides of 30 mg/mL (Zhang et al. 2008), suggesting superior thrombin inhibition efficiency.

**TABLE 3:** Thrombin inhibition rate of hemp seed peptides with varying molecular weight or concentration.

Concentration (g/mL)	Below 3 kDa (%)	Below 5 kDa (%)
0.002	9.32	3.58
0.004	36.55	11.51
0.006	63.33	38.39
0.008	78.74	51.58
0.010	99.38	62.44

**FIGURE 2:**
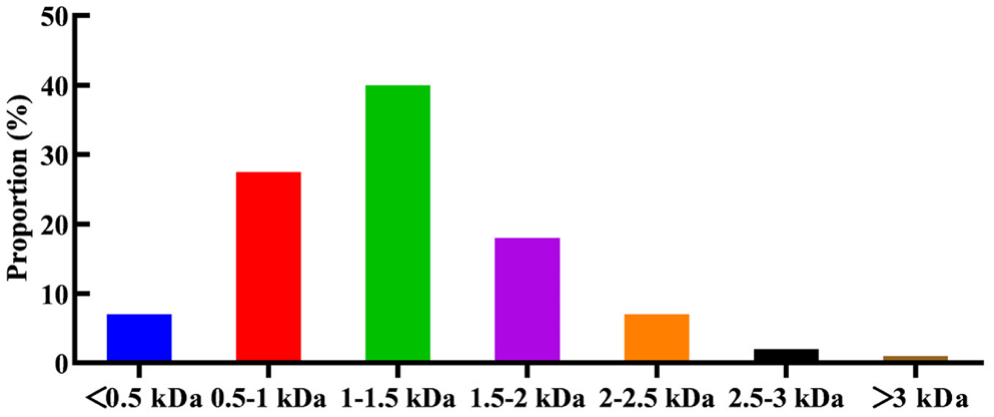
Mass distribution of hemp seed polypeptides.

Subsequently, the LC–MS/MS method was used to identify peptides with a molecular below 3 kDa. As shown in Figure 2, a total of 1741 peptide sequences were characterized, all containing fewer than 25 amino acids. Distribution analysis revealed that 40% of these peptides (677 sequences) were 1–1.5 kDa, primarily containing less than 13 amino acids. Additionally, peptides in the 0.5–1 kDa range constituted 27.5% of the total, corresponding to 302 sequences. Low molecular weight peptides with antithrombotic bioactivity usually contain 3–20 amino acid residues (Cheng et al. 2019).

It can be speculated that the hemp seed protein undergoes decomposition into smaller molecular peptides following enzymatic hydrolysis, and these smaller peptides exhibit enhanced antithrombotic activity. After hydrolysis, a few large polypeptides remain, potentially reducing antithrombotic activity.

Table 4 lists hemp seed peptides predicted to have antithrombotic activity based on molecular docking results. These candidates are nontoxic, nonallergenic, highly soluble, and show strong binding affinity (docking scores < −70). Additionally, the HemoPI tool was used to assess hemolytic activity, with a PROB score of 0 indicating a low likelihood of hemolysis. Notably, all peptides exhibited hemolysis scores of 0, suggesting they are unlikely to trigger hemolysis.

**TABLE 4:** Virtual screening results for candidate antithrombotic peptides.

Sequence	Mass	Allergenicity	PROB score	Toxicity	Solubility	Hydrophobicity	pI	Net charge	Docking score
KIQNQDDFRG	1219.6	No evidence	0	Nontoxin	Good	−1.900	5.96	0	−100.30
GRHSSENYR	1104.5	No evidence	0	Nontoxin	Good	−2.500	8.75	1.5	−96.99
GRGQDRELK	1057.6	No evidence	0	Nontoxin	Good	−2.267	8.75	1	−88.16
RASQEQLKA	1029.6	No evidence	0	Nontoxin	Good	−1.367	8.75	1	−87.77
VFSPSSQQTR	1135.6	No evidence	0	Nontoxin	Good	−0.920	9.72	1	−82.34
RADVIVVPAG	995.6	No evidence	0	Nontoxin	Good	1.070	5.84	0	−76.16
REQKTQQQV	1143.6	No evidence	0	Nontoxin	Good	−2.489	8.75	1	−75.38
VREPPQR	880.5	No evidence	0	Nontoxin	Good	−2.143	9.57	1	−72.73
GARFDER	849.4	No evidence	0	Nontoxin	Good	−1.686	6.07	0	−70.60
VTPEQNKQLK	1183.7	No evidence	0	Nontoxin	Good	−1.610	8.56	1	−70.25

*Note:* PROB score is the normalized SVM score and ranges between 0 and 1, i.e., 1 indicates high likelihood of hemolytic activity, and 0 indicates low likelihood.

### 3.2 MTC Detection Results

As shown in Table 5, zebrafish mortality reached 100% at hemp seed peptide concentrations ranging from 500 to 2000 μg/mL. Additionally, the phenotype of zebrafish exposed to 500 μg/mL of hemp seed peptide was observed to be worse than that of the model control group. Exposure to 250 μg/mL hemp seed peptide induced adverse effects in zebrafish, including prevalent loss of equilibrium and significantly suppressed locomotor activity relative to the model control group. However, at 125 μg/mL, zebrafish exhibited survival rates and locomotor activity comparable to controls, establishing this concentration as appropriate for efficacy evaluation. Given that, 125 μg/mL concentration for hemp seed peptide was selected as MTC for evaluating vascular endothelial injury.

**TABLE 5:** Toxic effects of different concentrations of hemp seed peptide on Albino zebrafish (*n* = 30).

Type	Concentration (μg/mL)	No. of dead zebrafish	Percentage (%)	Phenotype
Normal control	—	0	0	No obvious abnormality
Model control	—	0	0	No obvious abnormality
Hemp seed peptide samples	125	0	0	Similar to model control
	250	0	0	Worse than model control
	500	30	100	—
	1000	30	100	—
	2000	30	100	—

*Note:* The normal control group received no medication. The model control group was treated with 1.5 μg/mL punitinib. The hemp seed peptide groups were co‐treated with punitinib and corresponding concentrations of hemp seed peptide.

### 3.3 Hemp Seed Peptide Promoted the Quantity of Cardiac Erythrocytes in Zebrafish

**FIGURE 3:**
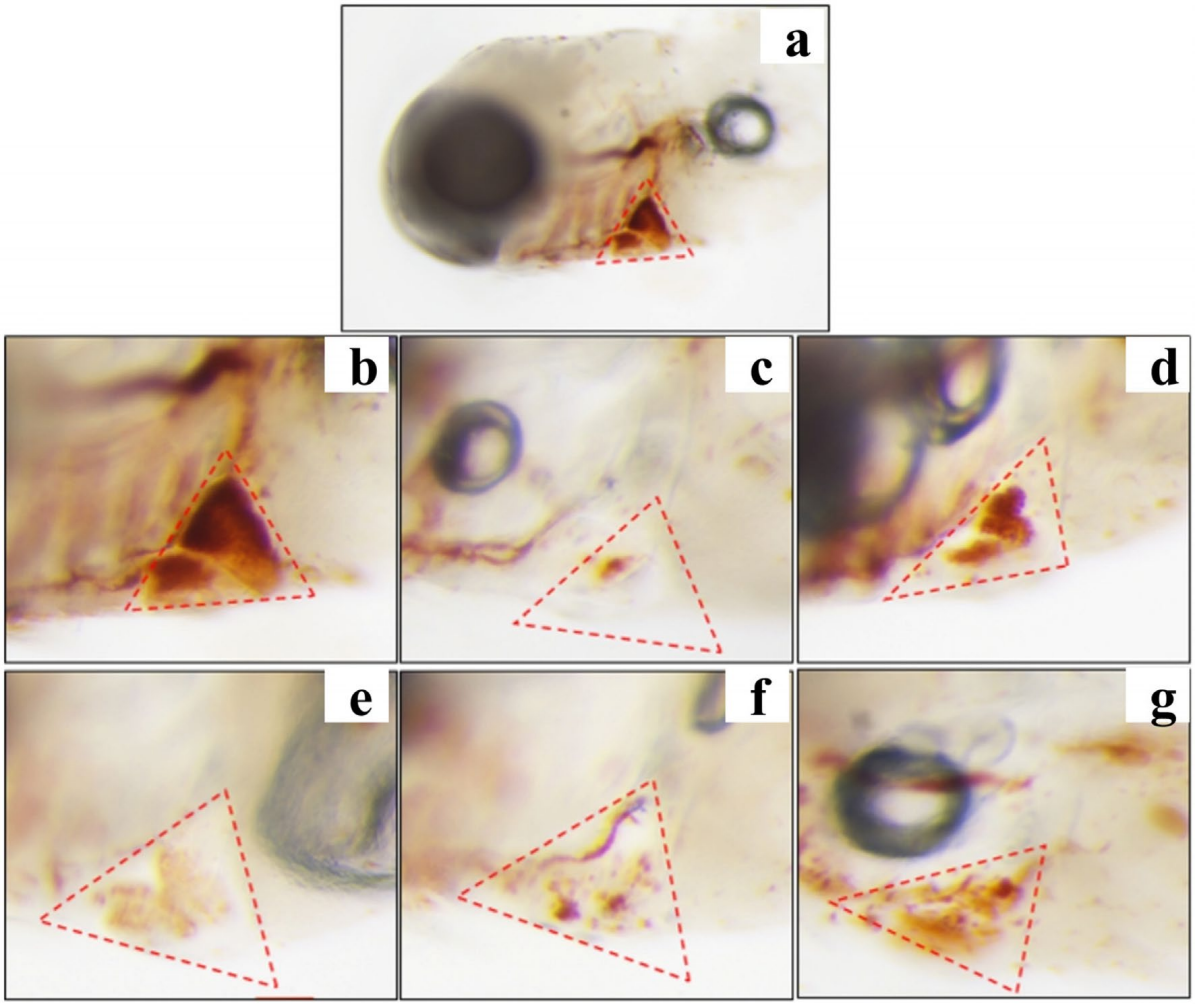
Typical diagram of albino zebrafish cardiac erythrocytes staining after treatment with hemp seed peptide or ponatinib. (a) Example diagram of erythrocytes staining intensity of zebrafish cardiac. (b) Normal control. (c) Model control. (d) Positive control. (e) Hemp seed peptide (31.25 μg/mL). (f) Hemp seed peptide (62.5 μg/mL). (g) Hemp seed peptide (125 μg/mL). The red dotted box indicates the analysis area in the zebrafish cardiac.

**FIGURE 4:**
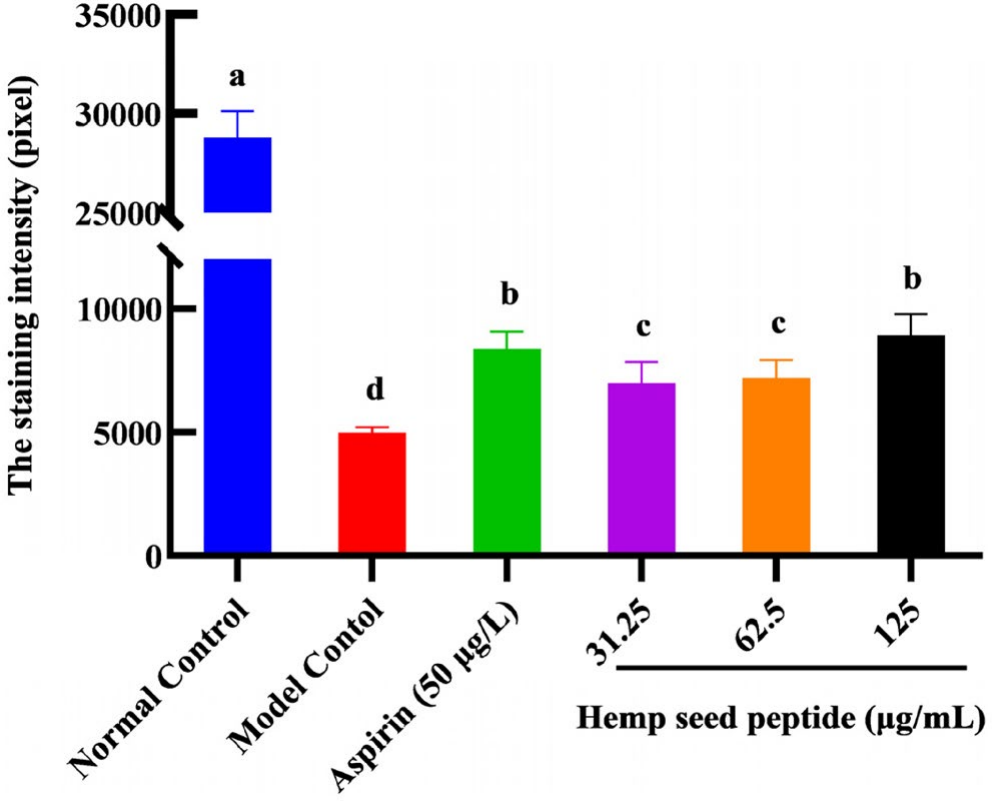
The staining intensity of erythrocytes in albino zebrafish cardiac after treatment with hemp seed peptide. Different letters indicate *p* < 0.05.

Thrombosis of the zebrafish tail vein can lead to a decrease in cardiac erythrocytes. Therefore, the erythrocyte staining intensity in the zebrafish's cardiac vein thrombosis region was measured to evaluate the antithrombotic activity of hemp seed peptide. As illustrated in Figures 3 and 4, an obvious decrease in staining intensity was observed in ponatinib‐treated zebrafish compared to the control group (*p* < 0.05), implying that ponatinib successfully induced a thrombosis model, which was consistent with the reported literature (Lin et al. 2023). As expected, the staining intensity of erythrocytes treated with three different concentrations of hemp seed peptide and aspirin showed an elevation (*p* < 0.05). Aspirin has been clinically proven to prevent thrombosis by inhibiting platelet aggregation and indirectly enhancing cardiac erythrocyte levels (Yu et al. 2024). Notably, the intensity in zebrafish treated with 125 μg/mL peptide exceeded that of the aspirin‐treated positive control group, but the differences were not statistically significant (*p* > 0.05).

Furthermore, the therapeutic efficacies for 31.25, 62.5, and 125 μg/mL hemp seed peptide were 8.40%, 9.27%, and 16.55%, respectively. These findings demonstrate that hemp seed peptide exhibits therapeutic potential against vascular endothelial injury‐associated thrombosis, as evidenced by the enhanced staining intensity of cardiac erythrocytes.

### 3.4 Hemp Seed Peptide Improved the Blood Flow Velocity in Zebrafish

**FIGURE 5:**
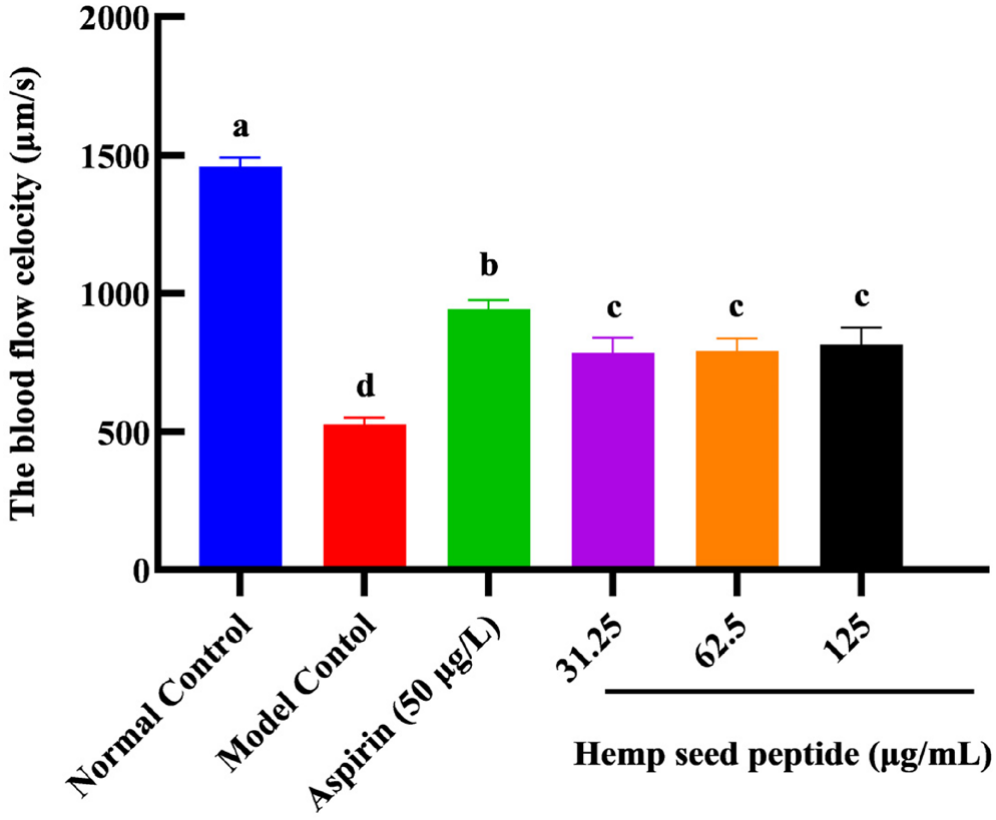
Comparison of blood flow velocity (μm/s) of AB wild‐type zebrafish in each group. Different letters indicate *p* < 0.05.

Thrombosis can restrict blood flow within the heart. The blood flow velocity of zebrafish was detected via microscopic observation. In the normal control group, it measured 1460 μm/s and remained stable (Figure 5). In contrast, the model control group showed a significantly reduced velocity to 527 μm/s (*p* < 0.05), indicating thrombosis formation, which is consistent with previous studies (Yu et al. 2024).

In the aspirin treatment group at 50 μg/mL, zebrafish exhibited a blood flow velocity of 943 μm/s, significantly higher than that of the model control group (*p* < 0.05), representing an increase of 78.9%. It illustrated that aspirin at this concentration could promote blood flow in thrombus‐induced zebrafish, demonstrating a certain degree of therapeutic effect.

For hemp seed peptide groups at concentrations of 31.25, 62.5, and 125 μg/mL, zebrafish exhibited blood flow velocities of 785, 792, and 816 μm/s, respectively, showing increases of 49.0%, 50.3%, and 50.3% compared to the model group (*p* < 0.05). Our findings indicated that all the above doses of hemp seed peptide could effectively enhance blood flow under experimental conditions and may provide varying degrees of preventive effects against ponatinib‐induced zebrafish thrombosis.

### 3.5 Hemp Seed Peptide Increased the Diameter of Thrombus Intersegmental Blood Vessels in Zebrafish

**FIGURE 6:**
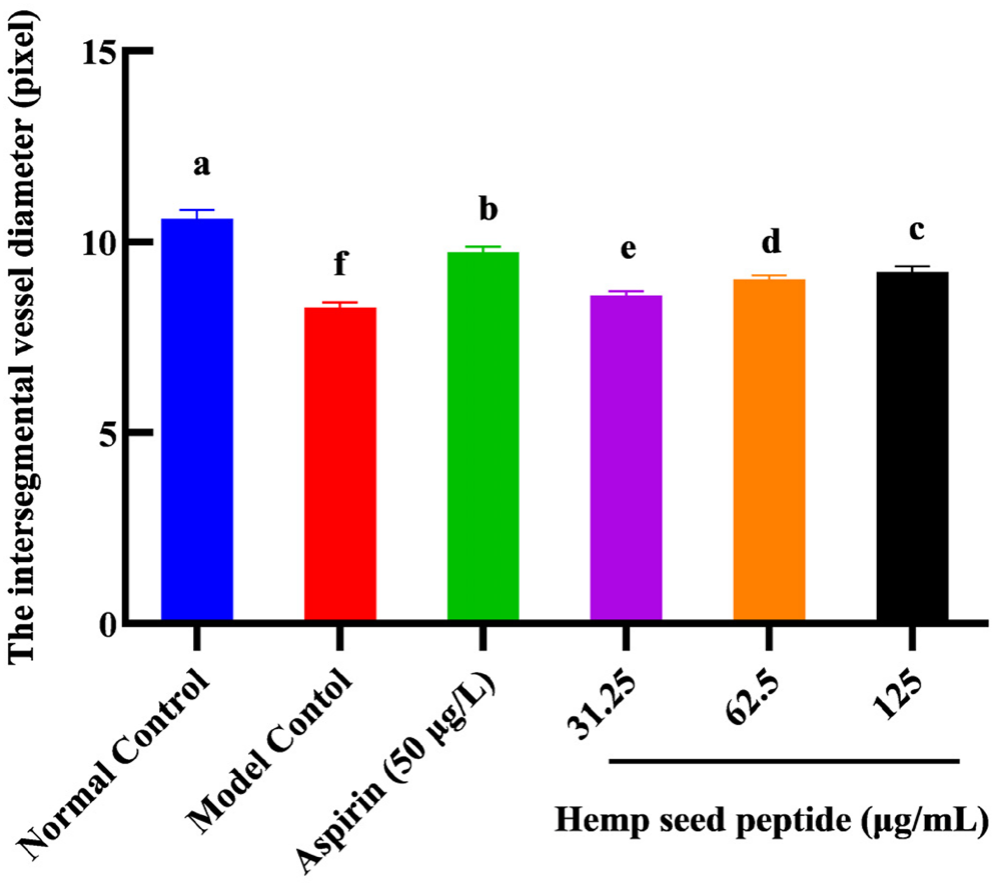
Comparison of intersegmental vessel diameter of *fli1*‐EGFP zebrafish in each group. Different letters indicate *p* < 0.05.

**FIGURE 7:**
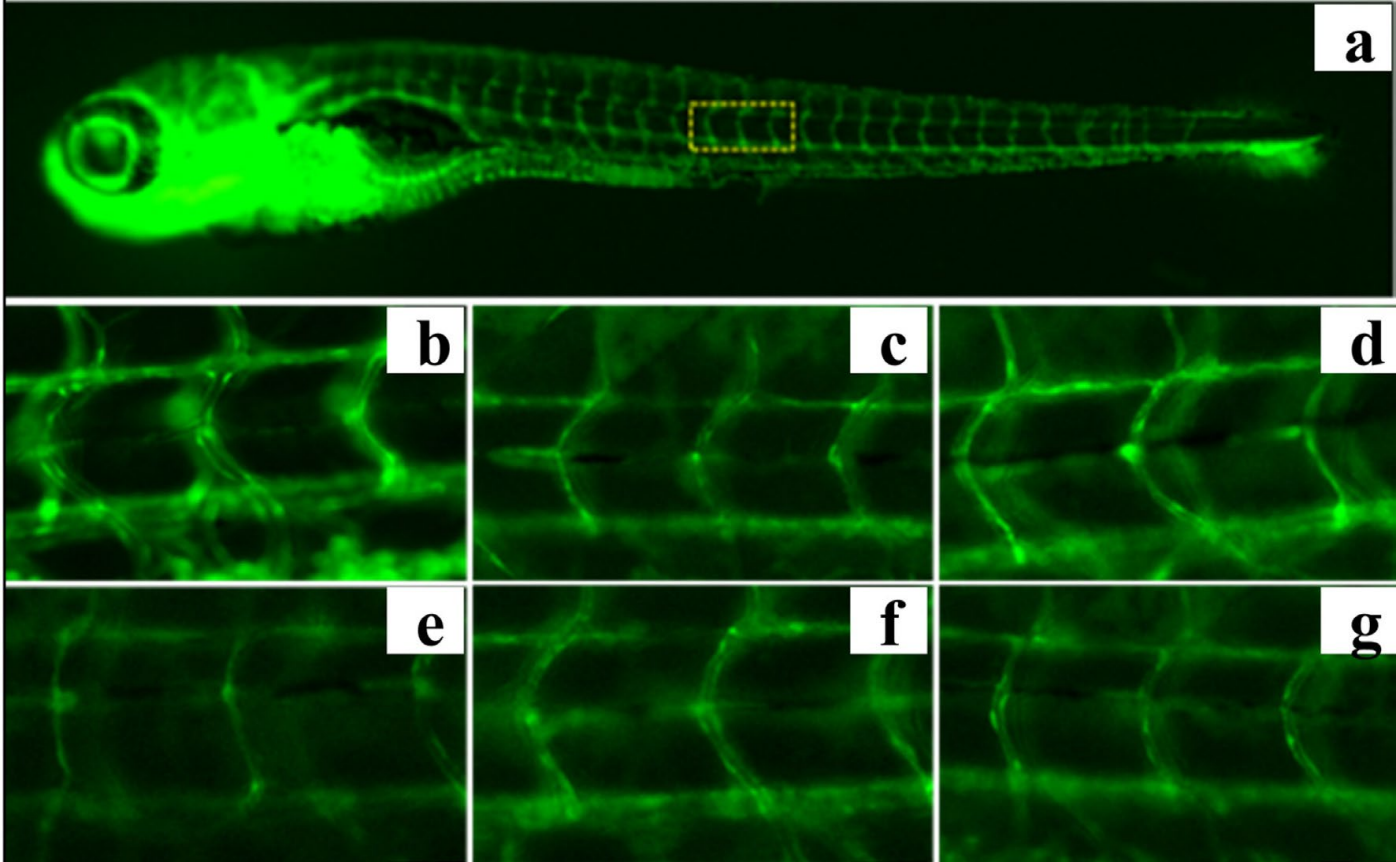
Typical diagram of intersegmental vascular diameter in *fli1*: EGFP zebrafish following treatment with hemp seed peptide or ponatinib. The yellow dotted box indicates the area of analysis for zebrafish intersegmental vascular (a) Example diagram of intersegmental vessel diameter analysis area of zebrafish. (b) Normal control. (c) Model control. (d) Positive contrast. (e) Hemp seed peptide (31.25 μg/mL). (f) Hemp seed peptide (62.5 μg/mL). (g) Hemp seed peptide (125 μg/mL).

Thrombus in the zebrafish trunk will reduce venous return, leading to decreased vessel diameter. To directly observe the change in blood vessel diameter, the transgenic green fluorescent vascular zebrafish (fli1‐EGFP) and NIS‐Elements D 3.20 advanced image processing software were used to analyze the diameter of zebrafish intersegmental vessels. Figures 6 and 7 demonstrated that ponatinib led to reduced blood vessel diameter. As the concentration of hemp seed peptide augmented, the average diameter of intersegmental vessels showed a dose‐dependent enlargement. In contrast to the model control, the zebrafish treated with 31.25, 62.5, and 125 μg/mL of hemp seed peptide for 18 h resulted in a notable improvement in intersegmental vessel diameter (*p* < 0.05), resulting in increases of 3.6%, 8.8%, and 11.2%. However, its efficacy is inferior to that of the positive control group (*p* < 0.05). This experiment proved that the hemp seed peptide improves the diameter of thrombus intersegmental blood vessels in zebrafish with vascular endothelial injury.

### 3.6 Hemp Seed Peptide Induced Vegfr1 and Inhibited Caspase‐3 Gene Expression in Zebrafish

*Vegfr1* is primarily expressed in vascular endothelial cells. Upon binding to VEGF, tyrosine residues within its intracellular signal domain undergo phosphorylation. This activation initiates downstream signaling cascades that drive endothelial proliferation, maturation, and ultimately contribute to neovascularization (Cai et al. 2017). In contrast, *caspase‐3* triggers programmed cell death in endothelial cells and upregulates platelet‐activating factor synthesis within these cells, thereby facilitating platelet aggregation (He, Wu, et al. 2024). The balance between *vegfr1*‐mediated vascular protection and *caspase‐3*‐induced endothelial apoptosis critically regulates thrombotic homeostasis, highlighting their opposing roles in antithrombotic actions. To further understand the mechanism involved in the antithrombotic effects of hemp seed peptides, we used RT‐qPCR to measure the mRNA expression levels of thrombus‐related genes *vegfr1* and *caspase‐3*.

**FIGURE 8:**
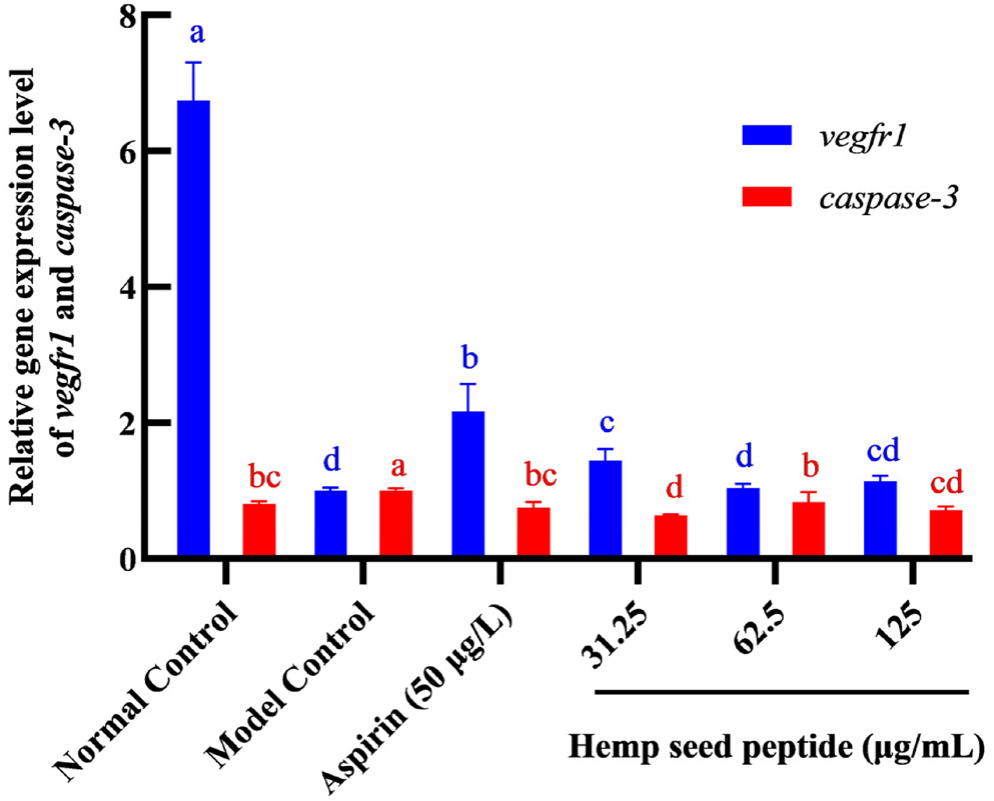
Comparison of relative expression of *vegfr1* gene and *caspase‐3* gene in each group. Different letters indicate *p* < 0.05.

As depicted in Figure 8, the relative mRNA expression level of *vegfr1* in the ponatinib‐stimulated group was significantly lower than that in the normal control group. At 62.5 and 125 μg/mL concentrations, hemp seed peptides could not elevate the expression of *vegfr1* in the MC group (*p* > 0.05). However, in the group treated with 31.25 μg/mL hemp seed peptide, the expression level of *vegfr1* mRNA was markedly higher than that in the model control group (*p* < 0.05), although it remained lower than that observed in the positive control group (*p* < 0.05). In addition, the results presented in Figure 8 showed that *caspase‐3* mRNA was highly expressed in the model control group, whereas treatment with aspirin could avoid it. Similar to aspirin, the upregulation induced by ponatinib was significantly reduced following treatment with 31.25, 62.5, and 125 μg/mL hemp seed peptide (*p* < 0.05). The relative gene expression levels in the 62.5 and 125 mg/mL peptide groups returned to normal levels (*p* > 0.05). In addition, the most significant inhibitory effect on the *caspase‐3* gene occurred at the peptide concentration of 31.25 μg/mL, showing a 36.8% inhibition rate. It was presumably due to lower peptide concentrations enhancing cellular uptake, leading to more potent caspase‐3 suppression.

Together, it was demonstrated that hemp seed peptide could upregulate *vegfr1* and downregulate c*aspase‐3* mRNA levels in the zebrafish thrombosis model.

## 4 Discussion

Recently, thrombotic diseases have surged to unprecedented levels, causing a serious threat to human health. In the present study, we utilized enzymatic hydrolysis technology to transform hemp seed processing by‐products, such as hemp seed cake, into peptides with antithrombotic activity. Our findings confirmed hemp seed peptides as potent antithrombotic agents that promote angiogenesis and inhibit intracellular apoptosis.

Antithrombotic activity evaluation techniques can be categorized into in vitro assays, in vivo models, and emerging in silico methods. Among in vitro techniques, thrombin inhibition assays serve as a fundamental tool for preliminary antithrombotic assessment due to their strong controllability and low cost. Nonetheless, these assays are inherently limited by their controlled experimental conditions, which fail to fully recapitulate the physiological complexity of human tissues. In vivo studies are particularly valuable for evaluating therapeutic interventions, as they provide critical insights into the physiological responses and efficacy of peptides within living organisms. While conventional peptide discovery remains limited by complexity, high costs, and labor intensity, animal models offer an indispensable and reliable platform to understand the underlying pathogenesis of thrombosis and assess the efficacy of novel antithrombotic peptides in preclinical studies (He, Du, et al. 2024). Currently, with the rapid advancement of bioinformatics, in silico analysis represents a cost‐effective and efficient approach for target identification of antithrombotic peptides. Some databases, such as ToxinPred, AllerCatPro, and Expasy, have been used as bioinformatics tools to provide structure and physical–chemical properties information about peptides. In addition, as a high‐throughput bioinformatics tool, molecular docking can not only predict the strength of protein‐peptide interactions, but also reveal possible binding mechanisms between ligands and receptors (Li et al. 2025). For instance, Ouyang et al. (2024) successfully utilized molecular docking to screen potential anticoagulant peptides in protein sequences from animals inhabiting deep‐sea hydrothermal vents and cold seeps. Similarly, Yang et al. (2025) identified six anticoagulant peptides from *Larimichthys crocea* protein using molecular docking in combination with in vitro evaluation. Nevertheless, molecular docking typically treats receptors as rigid, ignoring that both ligands and receptors can undergo conformational changes to achieve optimal binding (Yu et al. 2023). Therefore, the results of molecular docking may occasionally lack accuracy and require further validation. It is noteworthy that the main location of action for polypeptides with antithrombotic activity is still in the blood. Hence, the majority of these bioactive peptides require blood entry to function (Liu et al. 2023). An antithrombotic peptide may demonstrate promising bioactivity in vitro, but this does not guarantee similar effects will be observed in vivo due to the complexity and variability of biological systems (Toldra et al. 2020). Moreover, while these studies demonstrated strong in vitro thrombin‐inhibitory activity, they are limited by the lack of in vivo validation of peptide biological effects. Furthermore, industrial‐scale bioactive peptide preparation often faces challenges in scaling up and costly purification, which hinders commercialization (Cheng et al. 2024). In the present study, an integrated in vitro to in vivo approach provides a more comprehensive evaluation of enzymatic hydrolysate bioactivity.

The < 3 kDa fraction demonstrated remarkable thrombin inhibition (99.38% at 0.01 g/mL) and a low IC_50_ (4.55 mg/mL), outperforming rapeseed peptides of IC_50_ = 30 mg/mL (Zhang et al. 2008), scorpion‐derived peptides of IC_50_ = 23.65 mg/mL (Zhang et al. 2023) and egg peptides of IC_50_ > 50 mg/mL (Yang et al. 2007). This superiority is likely attributed to the unique peptide profile of hemp hydrolysates, where approximately 67% of identified sequences (0.5–1.5 kDa) fall within the optimal molecular weight range for antithrombotic bioactive peptide (Cheng et al. 2019). The complex composition of crude enzymatic hydrolysates may exert synergistic antithrombotic effects through multiple‐targeted mechanisms, such as inhibiting platelet aggregation, promoting fibrinolysis, and enhancing production of endothelial nitric oxide. These characteristics not only enhance therapeutic potential but also provide obvious advantages for reducing the difficulty of separation in industrial‐scale production. Moreover, using DOCK 6, we screened 10 representative hemp seed peptides, though their practical role and functional mechanisms require full elucidation. Future studies on their physicochemical and structural properties, along with the establishment of structure–activity relationships, will facilitate the rational design of novel targeted antithrombotic peptides.

The other primary purpose of this experiment was to investigate the antithrombotic activity of hemp seed peptide in vivo and its potential mechanism. Researchers have developed animal models like rats and mice, which are often costly, time‐consuming, and yield low success rates. Zebrafish share 87% genomic homology with humans. Hence, zebrafish have been well‐established as a vertebrate model for hematopoiesis and hemostasis research, widely used in high‐throughput drug screening (Sato et al. 2013). Furthermore, research has identified several key genes in zebrafish that significantly influence human thrombosis, demonstrating a notable degree of homology. Thrombosis models are commonly induced via microinjection or direct immersion using phenylhydrazine (PHZ), ferric chloride (FeCl_3_), arachidonic acid (AA), or ponatinib. PHZ, a strong oxidant, primarily induces thrombosis by causing oxidative damage to red blood cells, making this model particularly suitable for studying thrombosis associated with hemolytic anemia (Sato et al. 2013). Exogenous administration of AA triggers thrombosis via platelet aggregation in zebrafish (Luo et al. 2025), indicating its appropriateness for evaluating antiplatelet agents. Thrombosis involves tightly regulated processes including vasoconstriction, platelet activation, and blood coagulation, with endothelial cells playing a crucial role in the vascular response to injury leading to thrombus formation (Bochenek and Schafer 2019). Although FeCl_3_ induces thrombosis through direct chemical oxidation and damage to the vascular endothelium, this model is predominantly applied in murine studies (Li et al. 2016) and has not been widely adopted for zebrafish. In contrast, ponatinib, a tyrosine kinase inhibitor initially identified for its thrombotic side effects in leukemia treatment, causes endothelial dysfunction in zebrafish. It inhibits vascular endothelial proliferation, migration, and angiogenesis by blocking vascular endothelial growth factor receptor (VEGFR) signaling, ultimately leading to thrombosis (Hamadi et al. 2019). The ponatinib‐induced zebrafish thrombosis model is now utilized for in vivo thrombosis research and rapid screening/evaluation of antithrombotic drugs. For instance, He, Wu, et al. (2024) demonstrated that 1.5 μg/mL ponatinib treatment reduces blood flow in zebrafish larvae, inducing thrombosis, whereas Ye et al. (2020) employed this model to identify three dammarane‐type triterpenoids from *Rhus verniciflua* root with antithrombotic activity. An additional advantage of the ponatinib model is its pathological relevance, especially for mimicking thrombosis complications in leukemia patients undergoing therapy. We induced thrombosis in a zebrafish model using ponatinib, and our findings were consistent with previous results (Zhang et al. 2022). Ponatinib significantly reduced cardiac staining intensity by 43%, blood flow velocity by 177%, and vessel diameter by 21%. Nevertheless, the hemp seed peptide improved vascular endothelial injury, increasing the staining intensity of erythrocytes in the cardiac, enhancing the blood flow velocity, and expanding the diameter of intersegmental blood vessels. Notably, at 125 μg/mL, hemp peptides restored vascular function to near‐normal levels without inducing toxicity, suggesting favorable therapeutic prospects compared to heparin's bleeding risks (Li et al. 2020). In our study, the thrombosis model of zebrafish was used for the first time to verify the antithrombotic activity of hemp seed peptide, and the results showed that hemp seed peptide had significant antithrombotic activity. However, zebrafish models cannot fully replicate the complexity and variability of thrombosis in human patients (Griffin et al. 2024). Therefore, the specific clinical antithrombotic effects of hemp seed peptides require validation across multiple thrombosis models in the future. Data covering diverse thrombotic mechanisms would enhance the accuracy and robustness of our experimental findings.

Mechanistically, the observed antithrombotic effects align with the dual modulation of *vegfr1* and *caspase‐3*. Dose‐dependent improvements in hemodynamic parameters align with *vegfr1*‐mediated endothelial proliferation, analogous to Rubia cordifolia's pro‐angiogenic effects via VEGF‐A/kdrl upregulation (Chen et al. 2018). *Vegfr1* activation promotes endothelial cell proliferation and vascular repair (Griffin et al. 2024), while *caspase‐3* suppression mitigates apoptosis‐driven platelet aggregation (Raghunath et al. 2022). This dual‐action mechanism contrasts with single‐target approaches, such as direct thrombin inhibitors, potentially offering broader clinical applicability (Schwienhorst 2006). Intriguingly, lower peptide concentrations (31.25 μg/mL) maximally suppressed *caspase‐3*, possibly due to enhanced cellular uptake efficiency, whereas higher doses (125 μg/mL) optimized hemodynamic outcomes. This dose‐dependent divergence underscores the need for pharmacokinetic studies to balance bioavailability and bioactivity.

As is well known, thrombin drives platelet aggregation by activating platelets via PARs and GPIbα, enabling fibrinogen binding (De Candia 2012). It further promotes coagulation by activating Factor XIII (FXIIIa), which cross‐links fibrin to form a stable clot resistant to plasmin degradation (Souri et al. 2015; Zabczyk et al. 2021). Additionally, thrombin activates PAR1 on endothelial cells, reducing NO production and thereby facilitating platelet aggregation and thrombus formation (Li et al. 2022). Therefore, anticoagulant peptides may simultaneously possess platelet aggregation inhibitory activity, fibrinolytic properties, and the ability to promote NO secretion in endothelial cells. Among the 10 candidate peptides analyzed, two peptides—GARFDER and RASQEQLKA—demonstrate significant sequence similarity to known platelet aggregation inhibitors, suggesting potential shared inhibitory mechanisms. GARFDER contains a characteristic “FD” motif and exhibits a high density of acidic residues, closely resembling the oat‐derived antiplatelet peptide GEEFDAFTPK (Yu et al. 2016). Similarly, RASQEQLKA shares a conserved “QL” receptor‐binding motif and mixed charge profile with two established inhibitors, QLAQIPR and the scolopendra‐derived SQL peptide (Kong et al. 2013). These structural similarities indicate that GARFDER and RASQEQLKA may possess dual antithrombotic activities, combining thrombin inhibition with platelet aggregation suppression. Additional analysis identified two peptides—GRHSSENYR and RADVIVVPAG—that exhibit structural features associated with fibrinolytic activity. GRHSSENYR displays a hydrophobic‐polar “HSS” motif resembling the “TVAA”/“LLSY” sequences found in fibrinolytic peptides from Cordyceps militaris culture supernatant, along with a C‐terminal tyrosine residue that may enhance fibrin binding (Liu et al. 2015). RADVIVVPAG features a hydrophobic “IVV” core similar to the “VLL” region in known fibrinolytic peptides. These structural characteristics suggest potential roles in promoting fibrinolysis. Future research should focus on characterizing specific effects of these peptides on platelet aggregation pathways (particularly through PAR and GPIbα interactions), their fibrinolytic potential (via FXIIIa modulation), and their influence on endothelial nitric oxide production (through eNOS regulation). Such investigations would provide crucial insights into their therapeutic potential for antithrombotic applications, particularly in conditions requiring multi‐target intervention strategies. The combination of thrombin inhibition, antiplatelet activity, and fibrinolytic enhancement could offer novel approaches for developing more effective and safer antithrombotic therapies.

## 5 Conclusion

In summary, although preliminary, this study utilized zebrafish models to verify that hemp peptides exert antithrombotic effects via the dual regulation of vegfr1 upregulation and caspase‐3 downregulation, presenting a novel therapeutic strategy. By repurposing agricultural by‐products into value‐added nutraceuticals, it can effectively enhance farmers' income while utilizing dietary hemp peptides for disease prevention and health improvement.

## Author Contributions

**Jie Shi:** conceptualization (equal), funding acquisition (equal), writing – original draft (equal). **Jingyi Ge:** data curation (equal), writing – review and editing (equal). **Zhenghai Zhang:** investigation (equal), validation (equal). **Lianhui Wei:** resources (equal), software (equal). **Yan Dong:** methodology (equal), project administration (equal). **Guowei Li:** software (equal). **Qingli Yang:** supervision (equal). **Jing Pan:** validation (equal). **Yanru Ji:** project administration (equal), supervision (equal).

## Conflicts of Interest

The authors declare no conflicts of interest.

## Data Availability

The data that support the findings of this study are available from the corresponding author upon reasonable request.
